# Comparing efficacy of a sweep net and a dip method for collection of mosquito larvae in large bodies of water in South Africa

**DOI:** 10.12688/f1000research.8351.1

**Published:** 2016-04-21

**Authors:** Katherine K. Brisco, Anthony J. Cornel, Yoosook Lee, Joel Mouatcho, Leo Braack

**Affiliations:** 1 Mosquito Control Research Laboratory, Kearney Agricultural Center, Department of Entomology and Nematology, UC Davis, Parlier, CA, USA; 2 Vector Genetics Laboratory, Department of Pathology, Microbiology and Immunology, School of Veterinary Medicine, UC Davis, Davis, CA, USA; 3 Centre for Sustainable Malaria Control, Faculty of Health Sciences, University of Pretoria, Pretoria, South Africa

**Keywords:** larval sampling, Anopheles, Culex, sweep net, dipping

## Abstract

In this study we tested an alternative method for collecting mosquito larvae called the sweep net catch method and compared its efficiency to that of the traditional dip method. The two methods were compared in various water bodies within Kruger National Park and Lapalala Wilderness area, South Africa. The sweep net catch method performed 5 times better in the collection of *Anopheles* larvae and equally as well as the dip method in the collection of *Culex* larvae (p =8.58 x 10 ^-5^). Based on 15 replicates the collector’s experience level did not play a significant role in the relative numbers of larvae collected using either method. This simple and effective sweep net catch method will greatly improve the mosquito larval sampling capacity in the field setting.

## Introduction

Traditionally, the larval dip method, as described in detail below, has been the standard method for the collection and sampling of mosquito larvae ( O’Malley, 1995). However, this method of collection has proven unsatisfactory for the collection of large numbers of *Anopheles* mosquito larvae from large water bodies needed for our mosquito genetics studies. Many species of *Anopheles*, such as *An*. *funestus* ( Tuno *et al.*, 2007) and *An. coluzzii* ( Gimonneau *et al.*, 2015), readily dive making them difficult to collect from large bodies of water. Collecting *Anopheles* larvae therefore often means spending considerable time in the field. Also, the larval dip method requires significant experience in dipping techniques and source analysis skills in order to successfully collect the desired genus of larvae ( O’Malley, 1995), presenting challenges for a novice.

This limitation of the dipping method motivated us to evaluate a sweep net system similar to methods used by Trapido & Aitken (1953) and Robert *et al.* (2002) as an alternative approach for collecting larvae to increase catch numbers, especially of *Anopheles*, and reduce time spent collecting in the field. The method also had to be simple enough for a novice to successfully use. We named our modified sweep net approach the “sweep net catch (SNC)” method and tested it as described below.

## Methods

### Sweep net catch (SNC) method

A tray 5.7 cm deep × 45.7 cm in length × 31.8 cm in width (BioQuip ^®^Products, Rancho Dominguez, CA- catalogue number 1426c) or a tray similar in size was pre-filled halfway with the cleanest water available from the collecting site and set aside. The sweep net was essentially designed as a sieve consisting of a 25 cm diameter metal ring mounted to the end of a 1.2 meter pole. White nylon or cotton fabric of fine mesh (177.8 × 177.8 mesh per cm or less) was sewn onto the metal ring so water could be sieved through the net. The net was held at a 45° angle and pushed through the water ahead or next to the collector to avoid casting a shadow on the water surface. The top half of the net was above the water surface and the bottom half below and we walked at a slow pace. A visual representation of this process can be seen in the first half of the accompanying Video (time point 0:04 – 1:20 minutes). This process was continued for ten minutes per trial. During the ten minute period, the net was periodically inverted and its contents transferred to the tray containing pre-filled water. After completing ten minutes of sweeping, any mosquito larvae present in the plastic tray were picked out of the tray using a pipette (BioQuip ^®^Products, Rancho Dominguez, CA- catalogue number 4776) and set aside for further cleaning and storage.

### Dip method

A 350 mL dipper (BioQuip ^®^Products, Rancho Dominguez, CA- catalogue number 1132) attached to the end of an approximately 1.2 meter-long pole was used to scoop samples from the water. The collector then inspected the cup for the presence of mosquito larvae. If no larvae were present, the cup was emptied and the collector would try again in another nearby spot. If larvae were present, they were removed using a small pipette (BioQuip ^®^Products, Rancho Dominguez, CA- catalogue number 4776) and transferred to another holding cup prior to taking another dip sample. The collector would continue this process for ten minutes. A visual representation of this process can be seen in the second half of the accompanying Video (time point 1:21 – 2:23 minutes).

### Collection sites

Larval collections took place in pools along the Shingwedzi River (23.11604°S; 31.37524°E) and at Lake Panic in Skukuza (24.98472°S; 31.5797°E) in the Kruger National Park, South Africa, and along the shores of a lake in the Lapalala Wilderness area (23.90125°S; 28.29387°E) in the Limpopo Province, South Africa. Five replicate trials were performed along the Shingwedzi River, four trials in Skukuza, and six trials in Lapalala. For each trial the dip method was performed by one collector and the SNC method was performed by another, resulting in fifteen replicates of each method. Collectors, ranging in experience level, alternated collection methods they performed between sites.

All larvae collected were separated by genus and counted. The method used for collection (dip method or SNC method) as well as the collector’s name and experience level (experienced – having dipped for larvae before or novice – having never dipped for larvae before) were also recorded. Cornel and Braack were considered experienced collectors and all others were novices who had never dipped for larvae before. The raw data used for data analysis is available (see Data availability).

### Data analysis

**Figure 1.**
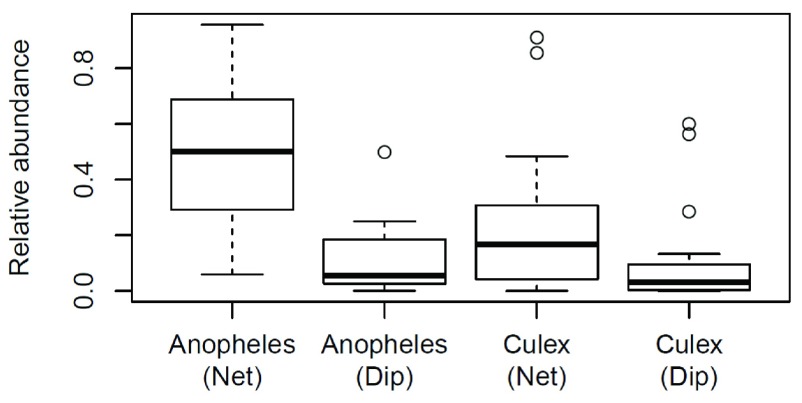
Box plot representations of relative abundance of mosquito specimens grouped by genus per collection. Each collection constitutes a ten minute long sweep net and a ten minute long dip.

**Figure 2.**
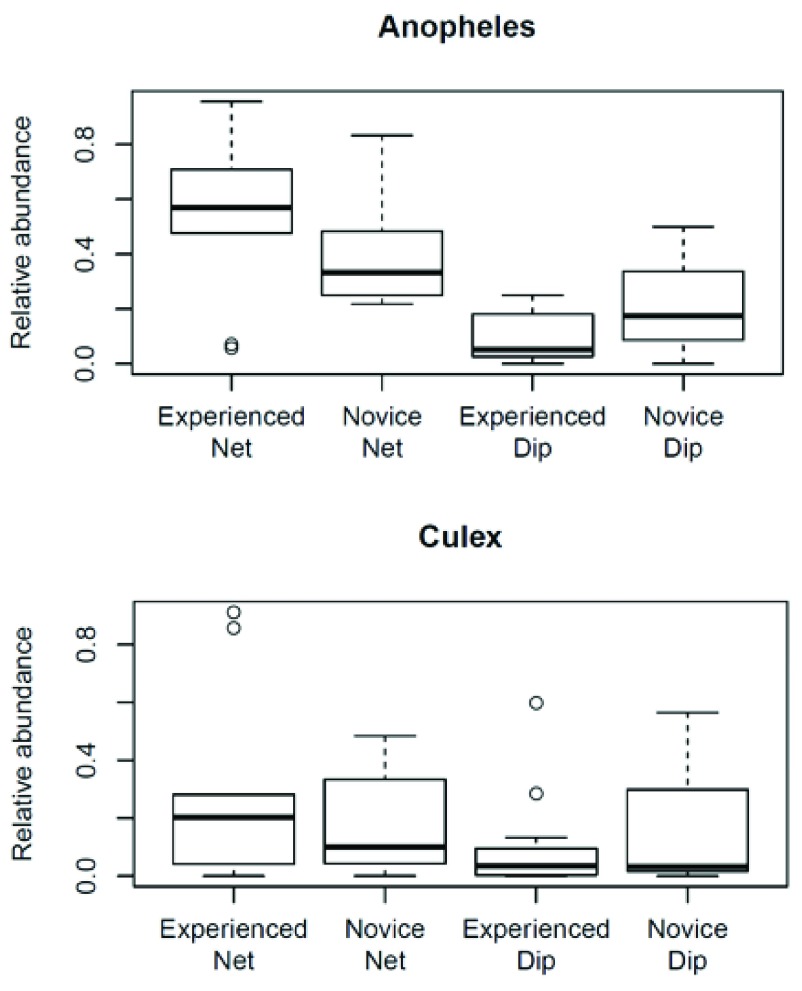
Box plot representations of relative proportions of mosquito specimens grouped by genus captured using the larval sweep net and larval dip by collectors of different experience levels.

A relative abundance of mosquito specimens grouped by genus per collection was calculated. The Wilcoxon-Rank-Sum test implemented in the R statistical package version 3.0.0 was used to compare the efficacy of the two collection methods ( Figure 1) and to see if experience level of collectors was a contributing factor in the relative proportions of each genus collected using each collection method ( Figure 2). 

## Results

As shown in Table 1, a total of 99 *Anopheles* larvae were collected using the dip method, of which 77 were from Shingwedzi, 4 from Skukuza, and 18 from Lapalala. A total of 605 *Anopheles* larvae were collected using the SNC method, of which 530 were from Shingwedzi, 20 from Skukuza, and 55 from Lapalala. A total of 61 *Culex* larvae were collected using the dip method, 22 being from Shingwedzi, 8 from Skukuza, and 31 from Lapalala. A total of 176 *Culex* larvae were collected using the SNC method, 63 of these from Shingwedzi, 85 from Skukuza, and 28 from Lapalala.

**Table 1.** Total number of larvae collected per genus per collection method at each location.

	*Anopheles*		*Culex*	
Location	Dip method	Sweep net method	Dip method	Sweep net method
**Shingwedzi**	77	530	22	63
**Skukuza**	4	20	8	85
**Lapalala**	18	55	31	28
	99	605	61	176

The number of collected larvae were highly variable between trials, reflecting the general larval density variation between sites. To control for this site variation and compare the relative performance of each collection method, a relative proportion of each genus collected per trial was calculated. The Wilcoxon-Rank-Sum tests on the relative abundance data showed ( Figure 1) the SNC method (mean relative abundance = 0.51 ± 0.28) performed significantly better than the dip method (mean relative abundance = 0.12 ± 0.13) in the collection of *Anopheles* larvae (Wilcoxon-Rank-Sum test P = 8.58 × 10 ^-5^). There was no significant difference in the collection of *Culex* larvae between the SNC method (mean relative abundance = 0.25 ± 0.29) and the dip method (mean relative abundance = 0.12 ± 0.20) (Wilcoxon-Rank-Sum test P = 0.050).

The collector’s experience was not a contributing factor in the relative proportions of each genus collected using either the SNC method or the dip method (Wilcoxon-Rank-Sum test P ≥ 0.21) ( Figure 2).

Visual representation of both the sweep net catch and dip larval collection methods as performed by Braack and Cornel. The SNC method can be seen in the first half of this video (0:04 - 1:20 minutes) while the Dip method can be seen in the second half (1:21 - 2:23 minutes). Click here for additional data file. Copyright: © 2016 Brisco KK et al. 2016 Data associated with the article are available under the terms of the Creative Commons Zero "No rights reserved" data waiver (CC0 1.0 Public domain dedication).

Raw larval collection data for all 15 replicates, including the relative abundance of Anopheles and Culex collected and the estimated time taken to collect one larva for each replicate. Includes: trial location, trap type, trap handler,  number of mosquitoes collected, proportions of mosquitoes collected and estimated total time. Click here for additional data file. Copyright: © 2016 Brisco KK et al. 2016 Data associated with the article are available under the terms of the Creative Commons Zero "No rights reserved" data waiver (CC0 1.0 Public domain dedication).

## Discussion

Many of the *Anopheles* larvae collected were reared to adults and identified using a morphological key ( Gillies & Coetzee, 1987) and molecular assays ( Koekemoer *et al.*, 2002; Lee *et al.*, 2014; Scott *et al.*, 1993) (see Supplementary material for methods). The species collected included *An. arabiensis, An. quadriannulatus, An. coustani, An. pretoriensis* and *An. funestus* group ( *An. parensis*, *An. rivulorum*, *An. leesoni* and an as yet undetermined species).

The SNC method performed on average five times better than the dip method in the collection of *Anopheles* larvae. The SNC samples a larger volume of water than the dip method which likely contributes to the higher catches of *Anopheles* larvae. Many species, such as *An. funestus*, *An. arabiensis* ( Tuno *et al.*, 2007) and *An. coluzzii* ( Gimonneau *et al.*, 2015), are known to dive and remain in the substrate for long periods of time when there has been a disturbance on the surface or for feeding purposes. The larger net diameter allows the increased capture of these larvae as they begin to submerge. In shallow parts of the water body the SNC also scoops along the substrate and may collect larvae that have dived and rested there. From our observations the SNC method disturbs the surface of the water less than dipping, which possibly reduces diving behavior.

**Figure 3.**
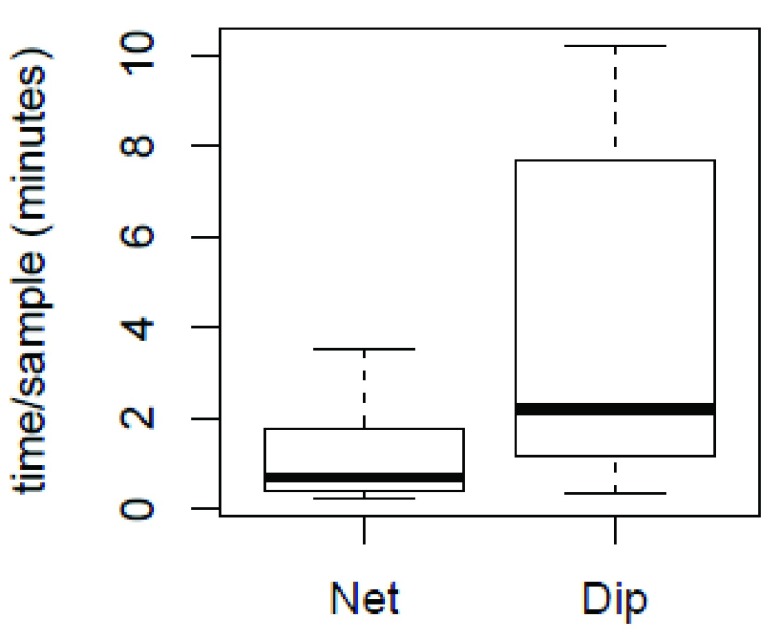
Box plot summary of processing time spent collecting larvae between the dip and sweep net collection methods.

More *Anopheles* may also have been captured using the SNC because collectors spent more time sieving through the water in the 10 minute period, thus covering a larger area, whereas during the 10 minutes of collecting, the dip method required spending time actively separating out the larvae after each dip. Depending on the water quality and larval density, the final larval separation for the SNC can be a time-consuming task as mud and debris can make it difficult to see the larvae in the tray. However, the overall processing time (collection and sample separation) per specimen for the SNC (1.09 ± 0.97 minutes) was 75% less than the dip method (4.17 ± 3.95 minutes) ( Figure 3). To calculate the average time spent collecting larvae with the SNC we added 12 seconds of processing time for each larva collected to the 10 minutes of sweeping for each trial. This time was then divided by the total number of larvae collected in that trial ( Data availability). The average time collecting larvae with the dip method was calculated by dividing the 10 minute dipping period by the total number of larvae collected for each trial ( Data availability).

Most of the *Culex* larvae collected were successfully reared to adults and identified using the key in Jupp (1996). The collections consisted of *Cx. poicilipes, Cx. simpsoni* and *Cx. neavei.* It has been shown that many species of *Culex* larvae exhibit significant diving behavior ( Workman & Walton, 2003), which suggests the SNC would also have increased efficiency in collecting these larvae as long as the surface was not too disturbed and the collector’s shadow was cast behind them. However, due to the relatively low numbers of *Culex* larvae collected throughout our study, we suspect there wasn’t a high enough population density of these larvae at any of our trial sites to accurately determine if either method was more efficient for the collection of *Culex* larvae. We recommend further research be conducted in areas where *Culex* larvae occur at higher densities to further evaluate whether the SNC performs better than the dip method.

Even though our current data does not show a significant difference in the relative proportion of larvae collected due to the collector’s experience, our personal observations suggest the SNC is a good method for novices to use. The SNC allows the inexperienced handler to easily collect high numbers of mosquito larvae without analyzing their technique or source characteristics as is required to be successful using the dip method ( O’Malley, 1995). A larger trial size may illuminate more conclusively if experience level does affect collection performance.

## Conclusion

We endorse and encourage the sweep net method as a preferred technique for larval collection that can be easily used in the field setting regardless of experience level. Our SNC method is particularly effective in capturing *Anopheles* mosquito larvae. The increased sensitivity of SNC towards *Anopheles* larvae may be due to (1) the sampling of a larger volume of water than the dipping cup, and/or (2) reducing disturbance of the water surface resulting in fewer larval dives. This increased sensitivity of the SNC method makes it an appropriate larval collection tool for studies when more accurate assessments of larval densities are required and when there is less time available to sample for larvae. In addition, the simplicity of the SNC method makes it a recommended choice for novice collectors. Further research is suggested to more rigorously test if a significant correlation between the collector’s experience level and the relative proportion of larvae collected by either method exists.

## Data availability

The data referenced by this article are under copyright with the following copyright statement: Copyright: © 2016 Brisco KK et al.

Data associated with the article are available under the terms of the Creative Commons Zero "No rights reserved" data waiver (CC0 1.0 Public domain dedication). 

*Figshare*: Visual representation of both the sweep net catch and dip larval collection methods as performed by Braack and Cornel. 10.6084/m9.figshare.3123274 ( Brisco *et al.*, 2016a).

*Figshare*: Raw larval collection data for all 15 replicates, including the relative abundance of *Anopheles* and *Culex* collected and the estimated time taken to collect one larva for each replicate. 10.6084/m9.figshare.3123463 ( Brisco *et al.*, 2016b).

## Supplementary material

Larval rearing methods.
