# Supplementary material for: Comparing efficacy of a sweep net and a dip method for collection of mosquito larvae in large bodies of water in South Africa
Source: F1000Res. 2016 Apr 21;5:713. [Version 1] doi: 10.12688/f1000research.8351.1 (PMC5390499; doi:10.12688/f1000research.8351.1)
Supplement: Supplementary file 3 [file f1000research-5-8980-s0002.tgz › 8fb322f1-28ec-42de-abb6-7cf5ecfdd696.docx]

**Methods**

Larval rearing and identification

Larvae collected were placed individually into 22 mm X 52 mm 5 dram plastic tubes (BioQuip®Products, Rancho Dominguez, CA – catalogue number 8905) one quarter filled with water from the collection site. A hole, approximately 15 mm in diameter, was cut in the center of the tube’s lid and a fine mesh netting was hot-glued over it. Larval and pupal skins were collected from each vial and preserved in 70% ethanol for slide mounting. Emerged adults, except for *Anopheles gambiae* complex and *Anopheles funestus* group were transferred individually to dry 5 dram plastic tubes identical to the ones previously described and allowed to die. They were then pinned and identified using morphological keys (Jupp 1996, Gillies and Coetzee 1987). All *An. gambiae* complex and *An. funestus* group adults were preserved alive in 70% ethanol and identified using molecular assays (Lee *et al.* 2014, Scott *et al.* 1993, Koekemoer *et al.* 2002).

**References**

Gillies MT, Coetzee M. **A supplement to the Anophelinae of Africa south of the Sahara.** The South African Institute for Medical Research Publishers, Johannesburg. 1987. ISBN 0620 10321 3.

Jupp PG. **Mosquitoes of Southern Africa.** Ekogilde Publishers, Hartebeespoort. 1996. ISBN 0.9583889-4-6.

Koekemoer LL, Kamau L, Hunt R, Coetzee M. **A cocktail polymerase chain reaction assay to identify members of the Anopheles funestus (Diptera: Culicidae) group.** *Am J Trop Med Hyg.* 2002, 6: 804-11.

Lee Y, Marsden CD, Nieman C, Lanzaro GC. **A new multiplex SNP genotyping assay for detecting hybridization and introgression between the M and S molecular forms of *Anopheles gambiae*.** *Mol. Ecol. Resour.* 2014 Mar; 14(2): 297-305. PMCID: PMC3947471.

Scott JA, Brogdon WG, Collins FH. **Identification of single specimens of the *Anopheles gambiae* complex by the polymerase chain reaction.** *Am J. Trop. Med. Hyg.* 1993; 49(4): 520-529.
