# Supplementary material for: Comparing efficacy of a sweep net and a dip method for collection of mosquito larvae in large bodies of water in South Africa
Source: F1000Res. 2016 Apr 21;5:713. [Version 1] doi: 10.12688/f1000research.8351.1 (PMC5390499; doi:10.12688/f1000research.8351.1)
Supplement: Peer review history

# Peer review: Referee response for version 1

**DOI:** 10.5256/f1000research.8980.r15220

**Published:** 2016-07-26

**Authors:** Norbert Becker

**Affiliations:** 1 German Mosquito Control Association, Institute for Dipterology, Speyer, Germany

The study design is appropriate and the article is well written. The use of a sweep net (SN) represents a significant improvement for the surveillance of developmental stages of anophelines. I know by experience how difficult and time consuming it is to assess the abundance of anopheles larvae especially in muddy water with a dipper. The use of a SN will allow a more precise and quick assessment of the larval density e.g. before and after larvicide treatments to calculate the mortality rates or to document the presence of larvae at all.

 I agree with the statements of the authors that the use of a sweep net is more efficient than the dip method when larvae of anophelines have to be counted. I agree also that the data concerning culicine larval counts are not sufficient to draw a conclusion of the efficiency of the SNC for the assessment of culicine larvae. You have also to take into consideration that in some programmes the standardized dip method is used to assess a threshold of larvae (e.g. 10 dips per breeding site) to start the control operation. I confirm that this research work fulfils all prerequisites for indexation and scientific standards.

I have read this submission. I believe that I have an appropriate level of expertise to confirm that it is of an acceptable scientific standard.

# Peer review: Referee response for version 1

**DOI:** 10.5256/f1000research.8980.r13506

**Published:** 2016-05-20

**Authors:** Wolfgang R Mukabana

**Affiliations:** 1 School of Biological Sciences, University of Nairobi, Nairobi, Kenya

The 'method article' by Brisco et al is well written, easy to read/understand and to the point. I have read the article keenly and with deep interest and on this basis declare that I have no reservations whatsoever against this article - its a simple but great piece of work.

 The Sweep Net Catch (SNC) method for collecting mosquito larvae described is long overdue for inclusion in tool kits for field entomologists.

 The video showing how larvae were sampled are clear. I note that the SNC method is carried out continuously for 10 minutes and gives it undue advantage over the dip method, which involves sampling with interruptions. However, the authors have discussed this issue adequately and in fact adjusted the length of sampling time for the dip method to legitimatize comparisons.

 Table 1 shows raw data of numbers of mosquito larvae collected by genus and sampling method. I suggest that the data presented be 'digested' a little further to show such important elements like number of sampling trials (N), mean mosquito catches, standard errors etc.

 The authors conclude that 'Our SNC method is particularly effective in capturing *Anopheles *mosquito larvae'. This statement is true given the numbers of larvae collected but looking at the video I'm inclined to think that the mosquito breeding sites selected for the studies were not typical habitats for culicine mosquito larvae, thus the use of the word 'particularly' is not due. In fact the authors state in their discussion section that "However, due to the relatively low numbers of *Culex* larvae collected throughout our study, we suspect there wasn’t a high enough population density of these larvae at any of our trial sites to accurately determine if either method was more efficient for the collection of *Culex* larvae."

I have read this submission. I believe that I have an appropriate level of expertise to confirm that it is of an acceptable scientific standard.
